# Alternative skeletal muscle index for sarcopenia diagnosis in elderly patients with type 2 diabetes mellitus: A pilot study

**DOI:** 10.3389/fendo.2023.1083722

**Published:** 2023-02-09

**Authors:** Lanyu Lu, Bowei Liu, Fuzai Yin

**Affiliations:** ^1^ Department of Endocrinology and Metabolic Diseases, Hebei Medical University, Shijiazhuang, Hebei, China; ^2^ Department of Endocrinology, The First Hospital of Qinhuangdao, Qinhuangdao, Hebei, China

**Keywords:** T2DM, sarcopenia, SMI, elderly, diagnosis

## Abstract

**Purpose:**

To determine an alternative skeletal muscle index (a-SMI), easy diagnosis of sarcopenia in elderly patients with type 2 diabetes mellitus (T2DM).

**Patients and methods:**

This cross-sectional study included 223 inpatients with T2DM (100 males, age range 60-89; 123 females, age range 60-87). Screening for grip strength and gait speed, measuring SMI by dual-energy X-ray absorptiometry (d-SMI) for sarcopenia diagnosis, according to the Asian Working Group for Sarcopenia (AWGS) 2019 consensus. The a-SMI was established by binary logistic regression analysis with positive screening population. To assess the conformance of the new diagnostic approach with the AWGS 2019.

**Results:**

Sarcopenia was present in 36.3% of the study population. 59 had normal d-SMI and 81 had low d-SMI in screening patients with probable sarcopenia. In univariate analyses for all positive screening population, body mass index (BMI), 25-hydroxyvitamin D (25 - (OH) VitD), high density lipoprotein cholesterol (HDL-C), hypertension (HTN), and gender were correlates of d-SMI. Binary logistic regression analysis revealed that male (*B* = 2.463, 95%*CI*: 3.640 ~ 37.883, *p* = 0.000), HTN (*B* = 1.404, 95%*CI*: 1.599 ~ 10.371, *p* = 0.003), BMI (*B* = -0.344, 95%*CI*: 0.598 ~ 0.839, *p* = 0.000), 25-(OH) VitD (*B* = -0.058, 95%*CI*: 0.907 ~ 0.982, *p* = 0.004) were independent factors for d-SMI detection. Based on the extracted four correlates, the a-SMI was determined. The area under receiver operating characteristic (ROC) curve was 0.842, sensitivity and specificity for the new diagnostic approach were 84.0% and 84.5%. In a statistical measure of agreement between the AWGS 2019 and the new diagnostic approach, the kappa coefficient was 0.669 (*p* < 0.001).

**Conclusion:**

The a-SMI - based on gender, obesity status, 25-(OH) VitD, and HTN history - can be used in the absence of the d-SMI to supplement the algorithm for sarcopenia diagnosis in elderly patients with T2DM.

## 1 Introduction

Type 2 diabetes mellitus (T2DM) is an important health condition in a growing ageing population, approximately 25% of people over the age of 65 years have diabetes (1). Patients with T2DM often have accelerated muscle loss and multiple comorbidities, such as hypertension (HTN); overweight/obesity; hyperlipidemia; stroke; chronic kidney disease (CKD); and cardiovascular disease (CVD) (1, 2). T2DM is characterized by insulin resistance, inflammation, increased advanced glycation end-products (AGEs), and oxidative stress (3). These characteristics may lead to losses in skeletal muscle mass, strength and function, accelerating the development of sarcopenia (3). The previous study reported, besides microvascular and macrovascular complications, sarcopenia had been described as a new complication in the elderly population with T2DM (4). Sarcopenia was an aging and disease-related syndrome characterized by progressive and generalized loss of skeletal muscle mass, low muscle strength or low physical performance, with the risk of falls, frailty, fractures, disability, hospitalization and death (5, 6).

In detecting sarcopenia, algorithms required measuring muscle strength or physical performance and skeletal muscle mass (5, 6). To date, several imaging modalities, including dual-energy X-ray absorptiometry (DXA), computed tomography (CT), magnetic resonance imaging (MRI), bioimpedance analysis (BIA) and ultrasound (US), had been developed to measure skeletal muscle mass and achieve the diagnosis of sarcopenia (7, 8). Recently, DXA has been often used to estimate muscle mass in clinical practice, and also been the only radiological tool with accepted cutoff values to diagnose sarcopenia (9). CT and MRI were considered the gold standards, but their application was mostly limited to research (7). Radiation exposure is a major limitation. And clinical CT protocols were not standardized across hospital sites. Segmentation of continuous whole body MRI is too cumbersome and expensive for clinical practice (8). US had been always regarded as a minor tool in sarcopenia, and BIA had its weakness in low precision especially in patients who have chronic illness (9, 10). However, these imaging modalities had caveats in terms of cost, possible radiation exposure, and limited accessibility for primary care and field studies.

Recently, Ken-ichiro Sasaki et al. reported that the equation based on sex, weight, and calf circumference could predict muscle mass in patients with CVD (11). Furthermore, a Japanese study developed a simple anthropometric equation, which incorporated sex, height, weight, waist circumference (WC), and calf circumference, to be potential as a reliable and an effective substitute for estimating skeletal muscle index (SMI) in the local community (12). At present, there are still many gaps in sarcopenia research, the European Working Group on Sarcopenia in Older People (EWGSOP) 2018 consensus recommended seeking accurate, affordable and simple muscle quality assessment tools, to facilitate early detection and better treatment of sarcopenia in clinical practice (5). Therefore, we aimed to determine an alternative skeletal muscle index (a-SMI) based on simple measurement indexes, and to validate the diagnostic value of new diagnostic approach for sarcopenia in elderly patients with T2DM.

## 2 Material and methods

### 2.1 Subjects

We performed a cross-sectional study in Chinese hospitalized patients over the age of 60 years with T2DM. The exclusion criteria included the following: 1) acute complications of diabetes mellitus such as diabetic ketoacidosis and hyperosmolar hyperglycemia; 2) acute myocardial infarction; acute cerebrovascular disease; acute inflammation; Gastrointestinal bleeding; Malignant tumor; 3) maintenance hemodialysis; 4) hepatic dysfunction (>3-fold elevation of alanine aminotransferase, aspartate aminotransferase); 5) severe osteoarthropathy or neuromuscular disease; 6) implantation of a pacemaker; 7) inability to understand/perform the exercise tests for this study; and 8) others judged ineligible by the investigators. This study was approved by the ethics committee of the First Hospital of Qinhuangdao. All subjects provided written informed consent before study initiation.

### 2.2 Diagnosis of sarcopenia

Sarcopenia was diagnosed by measuring skeletal muscle mass, muscle strength and physical performance according to the recommended diagnostic algorithm of the Asian Working Group for Sarcopenia 2019 consensus (AWGS 2019) (6). Sarcopenia was defined as low DXA-derived SMI (d-SMI) (< 7.0 kg/m^2^ in males; < 5.4 kg/m^2^ in females) associated with either low handgrip strength (< 28 kg in males; < 18 kg in females) or low gait speed (< 1.0 m/s) (6). Subjects were divided into sarcopenic and non-sarcopenic groups on this criterion.

### 2.3 Data collection

With the use of predesigned questionnaires, we collected the following patient data: general data such as age and gender, and comorbidities, as well as the results of DXA, biochemical and anthropometric measurements.

We diagnosed multiple diabetes comorbidities according to Standards of Medical Care in Diabetes—2014 (13). Common comorbid conditions included HTN, fractures and falls, macrovascular diabetes complications consisted of CVD, stroke, and peripheral artery disease (PAD), and microvascular complications included diabetic kidney disease, diabetic retinopathy and neuropathy (13).

Anthropometric measurements, including height, weight, and WC, were obtained while the subjects were in light clothing and not wearing shoes. Body mass index (BMI) was calculated by dividing weight (kg) by height squared (m^2^). A commonly used gait speed test is called the 6-m usual walking speed test, with speed measured manually with a stopwatch. We measured grip strength with the Jamar dynamometer (Performance health supply, inc., Cedarburg, WI, USA), and took the maximum reading of at least 2 trials using either both hands or the dominant hand with the same standard.

Peripheral venous blood samples were taken at 8:00 AM after at least 8-hours of fasting, and subjected to biochemical measurements, including fasting blood glucose (FBG), glycated hemoglobin (HbA1c), albumin (ALB), triglycerides (TG), cholesterol (TC), low density lipoprotein cholesterol (LDL-C), high density lipoprotein cholesterol (HDL-C), Cr-glomerular filtration rate (GFR), uric acid (UA), and 25-hydroxyvitamin D (25 - (OH) VitD).

Body composition included bone mineral density (BMD), total body fat, total body lean were measured by using DXA (MEDILINK SARL., France). The d-SMI was calculated as follows: the formula =the sum of the lean amount of the bilateral upper limbs and the bilateral lower limbs (kg)/height^2^ (m^2^).

### 2.4 Statistical analysis

Data were analyzed using SPSS (version 23.0 for Windows, SPSS Inc., Chicago, IL, USA). Continuous variables were expressed as mean (SD) and discrete variables were expressed as counts (percentages). Mutivariate logistic regression analysis was performed to determine independent factors for d-SMI, and established an alternative formula for d-SMI. The area under receiver operating characteristic (ROC) curve analysis was calculated to predict the diagnostic value of the a-SMI, and determined the cutoff value for a-SMI diagnosis. In a statistical measure of agreement between the AWGS 2019 and the new diagnostic approach based on a-SMI. Statistical significance was established at *p* < 0.05.

## 3 Results

### 3.1 Characteristics of sarcopenic and non-sarcopenic patients

As shown in **Table 1**, 81 of 223 patients (36.3%) were diagnosed as having sarcopenia in elderly patients with T2DM. Sarcopenic patients were older, and included higher numbers of males and subjects with HTN as compared with non-sarcopenic patients (*p* < 0.05). BMI, handgrip strength, gait speed, 25 - (OH) VitD, ALB, HDL-C, d-SMI, and BMD of right femoral neck, were significantly lower in sarcopenic patients than those in non-sarcopenic patients (*p* < 0.05).

**Table 1:** Baseline characteristics of sarcopenia and non-sarcopenic patients.

Variable	Non-sarcopenic group (n = 142)	Sarcopenic group (n = 81)	*t* or *χ* ^2^	*p*
Age ( years ) mean ( SD )	69.42 ( 5.67 )	71.30 ( 5.98 )	- 2.336	0.020
Male n ( % )	56 ( 39.4% )	44 ( 54.3% )	4.620	0.022
Height ( cm ) mean ( SD )	163.51 ( 7.76 )	164.59 ( 8.14 )	- 0.980	0.328
Weight ( kg ) mean ( SD )	68.64 ( 10.31 )	66.69 ( 10.59 )	1.347	0.179
BMI ( kg/m^2^ ) mean ( SD )	25.64 ( 3.14 )	24.52 ( 2.78 )	2.675	0.008
WC ( cm ) mean ( SD )	91.10 ( 9.71 )	92.14 ( 8.70 )	- 0.751	0.453
Handgrip strength ( kg ) mean ( SD )	24.76 ( 8.45 )	22.13 ( 7.79 )	2.297	0.023
Gait speed ( m/s ) mean ( SD )	1.00 ( 0.25 )	0.85 ( 0.23 )	4.440	0.000
SBP ( mmHg ) mean ( SD )	145.15 ( 18.13 )	145.20 ( 21.06 )	- 0.018	0.986
DBP ( mmHg ) mean ( SD )	81.74 ( 10.73 )	82.25 ( 12.36 )	- 0.322	0.748
T2DM duration ( years ) mean ( SD )	10.95 ( 8.97 )	13.12 ( 9.38 )	- 1.704	0.090
HTN duration ( years ) mean ( SD )	7.91 ( 10.61 )	8.53 ( 10.40 )	- 0.409	0.683
FBG ( mmol/L ) mean ( SD )	8.05 ( 3.17 )	8.47 ( 3.69 )	- 0.886	0.377
HbA_1_c ( % ) mean ( SD )	8.55 ( 2.19 )	8.87 ( 1.85 )	- 1.118	0.265
25-(OH) VitD ( nmol/L ) mean ( SD )	46.70 ( 13.62 )	41.26 ( 12.48 )	2.956	0.003
ALB ( g/L ) mean ( SD )	42.87 ( 3.94 )	41.12 ( 4.98 )	2.648	0.009
GFR ( mL/min/1.73m^2^ ) mean ( SD )	91.54 ( 17.58 )	89.23 ( 18.85 )	0.879	0.380
LDL-C ( mmol/L ) mean ( SD )	2.76 ( 0.83 )	2.87 ( 1.15 )	- 0.704	0.483
HDL-C ( mmol/L ) mean ( SD )	1.15 ( 0.24 )	1.03 ( 0.23 )	3.511	0.001
UA ( umol/L ) mean ( SD )	313.46 ( 87.74 )	309.60 ( 80.95 )	0.323	0.747
TG ( mmol/L ) mean ( SD )	1.80 ( 1.09 )	1.70 ( 1.04 )	0.710	0.479
TC ( mmol/L ) mean ( SD )	5.13 ( 1.26 )	5.03 ( 1.62 )	0.479	0.633
Total body fat ( kg ) mean ( SD )	34.48 ( 8.14 )	34.16 ( 7.23 )	0.298	0.766
Total body lean ( kg ) mean ( SD )	35.54 ( 5.88 )	34.47 ( 6.30 )	1.272	0.205
d-SMI ( kg/m^2^ ) mean ( SD )	6.11 ( 0.79 )	5.51 ( 0.80 )	5.343	0.000
BMD of right femoral neck ( g/cm^2^ ) mean ( SD )	0.77 ( 0.13 )	0.72 ( 0.16 )	2.113	0.036
BMD of left femoral neck ( g/cm^2^ ) mean ( SD )	0.76 ( 0.19 )	0.72 ( 0.15 )	1.484	0.139
BMD of L1-4 ( g/cm^2^ ) mean ( SD )	0.88 ( 0.21 )	0.87 ( 0.19 )	0.488	0.626
Diabetic retinopathy n ( % )	26 ( 18.6% )	14 ( 17.5% )	0.039	0.498
Diabetic neuropathy n ( % )	51 ( 36.2% )	38 ( 47.5% )	2.724	0.066
Diabetic nephropathy n ( % )	16 ( 11.3% )	13 ( 16.3% )	1.119	0.197
Stroke n ( % )	23 ( 16.2% )	13 ( 16.0% )	0.001	0.568
CVD n ( % )	36 ( 25.4% )	27 ( 33.3% )	1.621	0.132
Carotid plaque n ( % )	123 ( 87.9% )	73 ( 91.3% )	0.603	0.295
Lower limb arterial plaque n ( % )	122 ( 86.5% )	72 ( 88.9% )	0.261	0.387
HTN n ( % )	86 ( 60.6% )	60 ( 74.1% )	4.165	0.028
Falls n ( % )	24 ( 16.9% )	12 ( 14.8% )	0.166	0.418
Fractures n ( % )	8 ( 5.6% )	5 ( 6.2% )	0.027	0.542

Numerical data are expressed as mean **±** standard deviation (SD). For differences in proportions, the numbers in parenthesis denote the percentage.

BMI, body mass index; WC, waist circumference; SBP, systolic blood press; DBP, diastolic blood press; T2DM, type 2 diabetes mellitus; HTN, hypertension; FBG, fasting blood glucose; HbA_1_c, glycated hemoglobin; 25-(OH) VitD, 25-hydroxyvitamin D; ALB, albumin; GFR, Cr-glomerular filtration rate; LDL-C, low density lipoprotein cholesterol; HDL-C, high density lipoprotein cholesterol; UA, uric acid; TG, triglycerides; TC, cholesterol; d-SMI, DXA-derived skeletal muscle index; BMD, bone mineral density; L1-4, 1-4 lumbar vertebrae; CVD, cardiovascular disease.

### 3.2 The a-SMI in positive screening patients with probable sarcopenia

All participants were screened for handgrip strength and gait speed, and 140 positive screening population were found, which was a criterion in the first step to diagnose sarcopenia. 59 had normal d-SMI and 81 had low d-SMI in screening patients with probable sarcopenia, which was further diagnosed as sarcopenia. In univariate analyses for all positive screening population, BMI, 25 - (OH) VitD, HDL-C, HTN and gender were correlates of d-SMI (**Table 2**).

**Table 2:** Baseline characteristics of normal d-SMI and low d-SMI patients.

Variable	Normal d-SMI Group ( n=59 )	Low d-SMI group ( n=81 )	*t* or *χ* ^2^	*p*
Age ( years ) mean ( SD )	70.75 ( 6.07 )	71.30 ( 5.98 )	- 0.534	0.594
Male n ( % )	12 ( 20.3% )	44 ( 54.3% )	16.425	0.000
BMI ( kg/m^2^ ) mean ( SD )	26.56 ( 3.23 )	24.52 ( 2.78 )	4.000	0.000
WC ( cm ) mean ( SD )	92.89 ( 10.79 )	92.14 ( 8.70 )	0.416	0.678
FBG ( mmol/L ) mean ( SD )	8.01 ( 3.12 )	8.47 ( 3.69 )	- 0.771	0.442
HbA_1_c ( % ) mean ( SD )	8.81 ( 2.47 )	8.87 ( 1.85 )	- 0.166	0.868
25-(OH) VitD ( nmol/L ) mean ( SD )	46.42 ( 15.78 )	41.26 ( 12.48 )	2.160	0.033
ALB ( g/L ) mean ( SD )	42.28 ( 4.27 )	41.12 ( 4.98 )	1.411	0.161
GFR ( mL/min/1.73m^2^ ) mean ( SD )	88.37 ( 18.99 )	89.23 ( 18.85 )	- 0.256	0.799
LDL-C ( mmol/L ) mean ( SD )	2.74 ( 0.89 )	2.87 ( 1.15 )	- 0.678	0.499
HDL-C ( mmol/L ) mean ( SD )	1.17 ( 0.27 )	1.03 ( 0.23 )	3.261	0.001
UA ( umol/L ) mean ( SD )	319.1 ( 86.99 )	309.60 ( 80.95 )	0.659	0.511
TG ( mmol/L ) mean ( SD )	1.96 ( 1.14 )	1.70 ( 1.04 )	1.409	0.161
TC ( mmol/L ) mean ( SD )	5.15 (1.43 )	5.03 ( 1.62 )	0.438	0.662
Diabetic retinopathy n ( % )	9 ( 15.5% )	14 ( 17.5% )	0.095	0.473
Diabetic neuropathy n ( % )	20 ( 33.9% )	38 ( 47.5% )	2.584	0.076
Diabetic nephropathy n ( % )	8 ( 13.6% )	13 ( 16.3% )	0.192	0.425
Stroke n ( % )	13 ( 22.0% )	13 ( 16.0% )	0.808	0.248
CVD n ( % )	18 ( 30.5% )	27 ( 33.3% )	0.125	0.434
Carotid plaque n ( % )	52 ( 89.7% )	73 ( 91.3% )	0.100	0.486
Lower limb arterial plaque n ( % )	51 ( 86.4% )	72 ( 88.9% )	0.192	0.426
HTN n ( % )	30 ( 50.8% )	60 ( 74.1% )	8.021	0.004
Falls n ( % )	11 ( 18.6% )	12 ( 14.8% )	0.365	0.352
Fractures n ( % )	6 ( 10.2% )	5 ( 6.2% )	0.753	0.289

Numerical data are expressed as mean **±** standard deviation (SD). For differences in proportions, the numbers in parenthesis denote the percentage.

d-SMI, DXA-derived skeletal muscle index; BMI, body mass index; WC, waist circumference; FBG, fasting blood glucose; HbA_1_c, glycated hemoglobin; 25-(OH) VitD, 25-hydroxyvitamin D; ALB, albumin; GFR, Cr-glomerular filtration rate; LDL-C, low density lipoprotein cholesterol; HDL-C, high density lipoprotein cholesterol; UA, uric acid; TG, triglycerides; TC, cholesterol; CVD, cardiovascular disease; HTN, hypertension.

The dependent variable was whether positive screening population had lower d-SMI (normal d-SMI = 0, lower d-SMI = 1), and the independent variables were gender (female = 0, male = 1), 25- (OH) VitD, HTN (without = 0, with =1), BMI, HDL-C. As shown in **Table 3**, logistic analysis showed that male (*B* = 2.463, 95%*CI*: 3.640 ~ 37.883, *p* = 0.000), HTN (*B* = 1.404, 95%*CI*: 1.599 ~ 10.371, *p* = 0.003), BMI (*B* = -0.344, 95%*CI*: 0.598 ~ 0.839, *p* = 0.000) and 25 - (OH) VitD (*B* = -0.058, 95%*CI*: 0.907 ~ 0.982, *p* = 0.004) were independent factors for d-SMI. The HDL-C (*B* = -1.639, 95%*CI*: 0.035 ~ 1.090, *p* > 0.05) was not introduced in this study. Based on the extracted four correlates, a calculation formula for getting an a-SMI, which was the criterion in the second step to diagnose sarcopenia, was determined as follows: a-SMI = 2.463 x gender {(female = 0) or (male = 1)}+ 1.404 x HTN {(without = 0) or (with = 1)}+ {- 0.344 x (BMI, kg/m^2^)}+ {-0.058 x (25 - (OH) VitD, nmol/L)}+ 11.738

**Table 3:** Stepwise multivariate binomial logistic regression analysis for d-SMI.

Variable	Partial regression coefficient	*OR*	95%*CI*	*p*
Gender ( female = 0, male = 1)	2.463	11.743	3.640 ~ 37.883	0.000
25-(OH) VitD ( nmol/L )	- 0.058	0.944	0.907 ~ 0.982	0.004
HTN ( without = 0, with = 1 )	1.404	4.072	1.599 ~ 10.371	0.003
BMI ( kg/m^2^ )	- 0.344	0.709	0.598 ~ 0.839	0.000
HDL-C ( mmol/L )	- 1.639	0.194	0.035 ~ 1.090	0.063
Constant term	11.738	125191.792		0.000

Dependent Variable: d-SMI. Independent Variables: Gender, 25-(OH) VitD, HTN, BMI, and HDL-C.p < 0.05 was considered statistically significant.

d-SMI, DXA-derived skeletal muscle index; 25-(OH) VitD, 25-hydroxyvitamin D; HTN, hypertension; BMI, body mass index; HDL-C, high density lipoprotein cholesterol.

**Figure 1:**
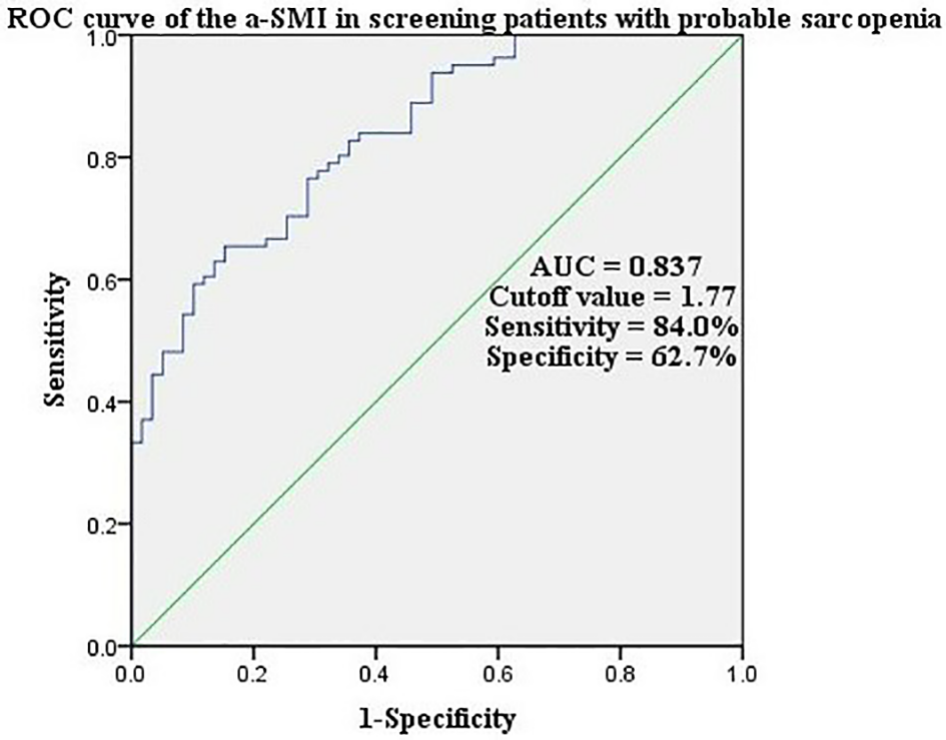
ROC curve of the a-SMI in screening patients with probable sarcopenia.

The a-SMI, obtained from the diagnostic regression formula, had a high accuracy in ROC curve analysis (sensitivity 84.0%, specificity 62.7%) when the area under ROC curve was 0.837 and the cutoff value for a-SMI diagnosis was 1.77 (**Figure 1**).

### 3.3 The new diagnostic approach and conventional diagnostic criteria for sarcopenia in elderly patients with T2DM

**Figure 2:**
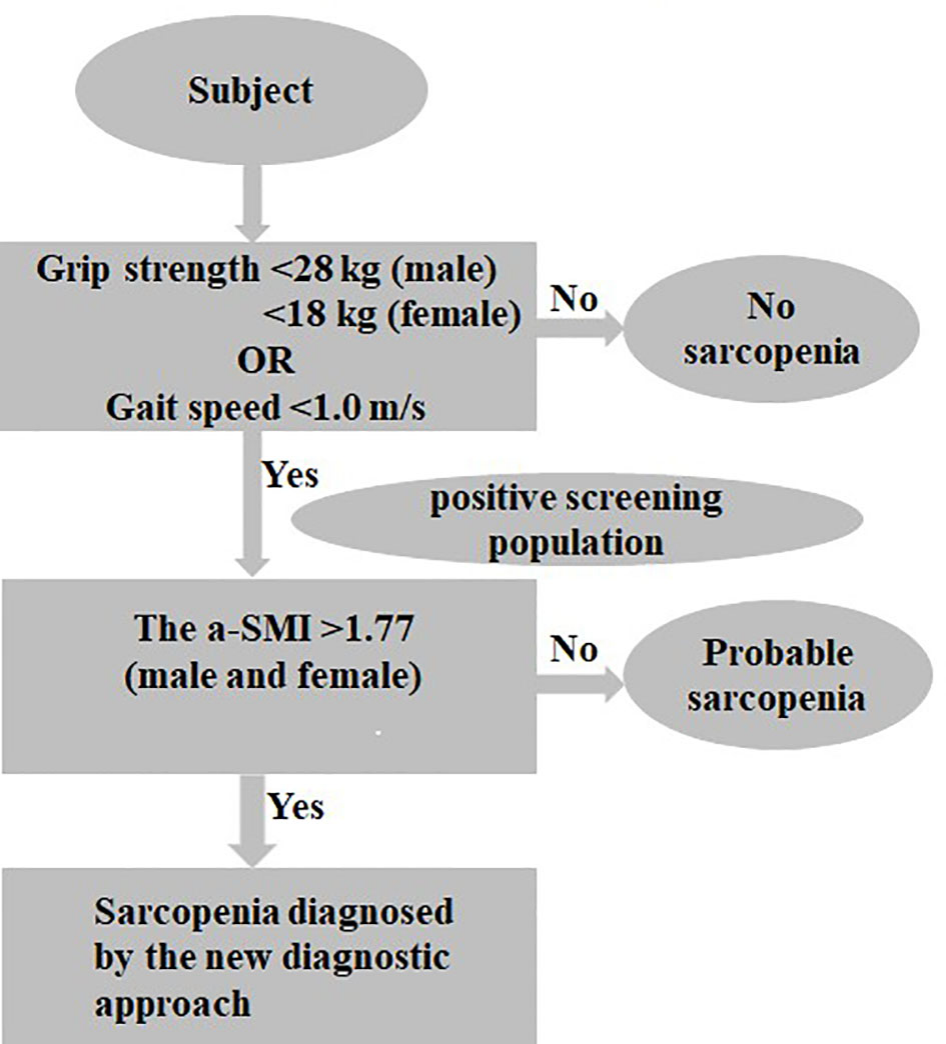
The new diagnostic approach for sarcopenia.

**Figure 3:**
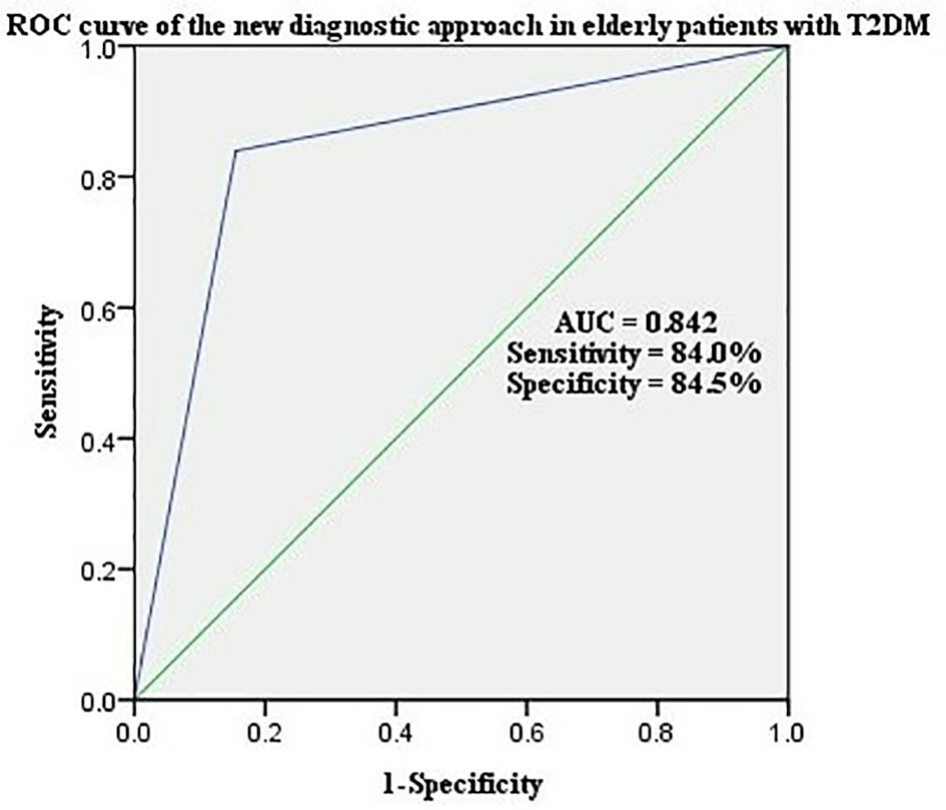
ROC curve of the new diagnostic approach in elderly patients with T2DM.

The a-SMI was the second step in the new diagnostic criteria for sarcopenia (**Figure 2**). The ROC curve was adopted to analyze the predictive value of the new diagnostic approach for sarcopenia in elderly patients with T2DM. The results showed that the area under ROC curve was 0.842, sensitivity was 84.0%, and specificity was 84.5% (**Figure 3**).

Lastly, in a statistical measure of agreement between the AWGS 2019 and the new diagnostic approach for all participants, the kappa coefficient was 0.669 (*p* < 0.001), indicating the two criteria were closely agreed (**Table 4**).

**Table 4:** In a statistical measure of agreement between the AWGS 2019 and the new diagnostic approach for all participants.

	Sarcopenia diagnosed by the new diagnostic approach			Sum
	No		Yes	
Sarcopenia diagnosed by AWGS 2019	No	n = 120 ( 84.5% of 142 )	n = 22 ( 15.5% of 142)	n = 142
	Yes	n = 13 ( 16.0% of 81 )	n = 68 ( 84.0% of 81 )	n = 81
Sum	n = 133		n = 90	n = 223

AWGS, the Asian Working Group for Sarcopenia.

## 4 Discussion

In the current study, we proposed a model to estimate d-SMI in positive screening population, which was calculated by a regression formula including the values of the subject’s sex, BMI, 25 - (OH) VitD, and HTN history, based on stepwise multivariate binomial logistic regression analysis. The new diagnostic approach developed in this study, was composed of handgrip strength, gait speed, and the a-SMI, could be used to supplement the algorithm for sarcopenia diagnosis in elderly patients with T2DM. For the diagnosis of sarcopenia, the prevalence of sarcopenia diagnosis by the new diagnostic method was 40.4%, and that was present in 36.3% according to the recommended diagnostic algorithm of the AWGS 2019. This indicated that the two criteria were in close agreement. And the a-SMI held potential as a reliable and an effective substitute in the absence of the d-SMI.

In our equation, a higher BMI level was associated with lower d-SMI. Previous studies reported that underweight subjects were at a higher risk of low skeletal muscle (14). A retrospective cross-sectional study included community-dwelling adults over 60 years of age suggested that obesity might have a protective effect in sarcopenic individuals (15). Several explanations had been proposed as to why obesity is not harmful to older adults (15). Overweight or obesity might be associated with a better supply of vitamins and other nutrients that ensure proper functioning (15). In addition, a higher BMI was associated with greater BMD, it might provide against osteoporotic fractures (16). Another important benefit of obesity in older adults was its association with higher levels of estrogen in this population (15). The estrogen produced by adipose tissue might exert protective effects on various organs through the effect of secretion. A Japanese study found elderly diabetes patients with low BMI and high body fat mass may be more likely to develop sarcopenia (17). Without taking body fat into account, subjects with high BMI tend to have more lean body mass (18, 19). Therefore, BMI might reflect lean mass in elderly diabetes patients (17).

Vitamin D played an important role in bone mineralization and musculoskeletal health (20). Our results had shown that 25 - (OH) VitD was significantly decreased in elderly patients with type 2 diabetic sarcopenia, and it was independent risk factor for d-SMI. It implied that 25 - (OH) VitD may play an important role in sarcopenia. The results of our study were in line with other studies where 25 - (OH) VitD was associated with muscle mass and function, increased the risk of sarcopenia in the elderly (21–23). A Korea study reported that sarcopenia was significantly associated with hypertension, particularly in the subjects with DM, and revealed that appendicular muscle mass (ASM) was independently related to SBP (24). HTN history in our study had adverse effects on d-SMI. Four possible mechanisms might explain the link between ageing muscle and HTN, including insulin resistance, inflammation, the relative paucity of myokines and alterations within the renin-angiotensin-aldosterone system (RAAS) (24–27).

Anagnostis et al. reported that the risk of sarcopenia were increased 1.72 -fold (95% *CI*: 1.1 ~ 2.69; *p* = 0.017) in male and 1.46 -fold (95% *CI*: 0.94 ~ 2.25; *p* = 0.08) in women with T2DM, and this difference between genders was not significant (28). In this study, the prevalence of sarcopenia in the T2DM patients aged 60 years was 19.7% for males and 16.6% for females. Males in our study had adverse effects on d-SMI. Testosterone levels in men decline with age, adversely affecting the distribution of muscle and adipose tissue (29). Earlier study had shown that men have more lean mass, and women have more fat mass. As people get older, men were more likely to accumulate adipose tissue around the trunk and abdomen, while women usually accumulate adipose tissue around the hips and thighs (30).

For the diagnosis of sarcopenia, it was necessary to measure grip strength, gait speed, and d-SMI. Grip strength and gait speed can be easily measured with small and simple instruments. We propose the a-SMI -based on gender, obesity status, 25-(OH) VitD, and HTN history - could be used in the absence of the d-SMI. Sarcopenia was diagnosed by the cutoff value of the a-SMI. Compared with MRI, CT and DXA, a-SMI can be performed in a small infirmary at a lower cost without radiation hazards. We did not define formulas by sex because we did not think it appropriate to define formulas based on data from a small number of men and women with sarcopenia. Nonetheless, the accuracy of the diagnosis of sarcopenia needs to be verified with surrogate indices and cut-off values in other populations.

There were some limitations in our study. First, due to the absence of CT and MRI (gold standards) for sarcopenia diagnosis, DXA recommended by AWGS 2019 was used in this study. The second concerns the formula, which calculated an abstract dimensionless proxy with quite different cut-off values, did not estimate muscle mass in kg or not a SMI in kg/m^2^. Third, it was a single-center study with a small population, limiting the ability to apply the result to the other ethnic groups, which needs to be further verified in other groups and institutions.

## 5 Conclusion

The present study demonstrates that the a-SMI - based on gender, obesity status, 25-(OH) VitD, and HTN history - can be used in the absence of the d-SMI to supplement the algorithm for sarcopenia diagnosis in elderly patients with T2DM. We believe that this new diagnostic approach of sarcopenia may be a useful tool for early prevention and early treatment of elderly T2DM patients with sarcopenia, thereby mitigating the adverse outcomes of T2DM patients due to sarcopenia.

## Data availability statement

The raw data supporting the conclusions of this article will be made available by the authors, without undue reservation.

## Ethics statement

This study was approved by the ethics committee of the First Hospital of Qinhuangdao. All subjects provided written informed consent before study initiation.

## Author contributions

BL: Conceptualization, Funding Acquisition, Resources, Supervision, Writing - Review & Editing. LL: Conceptualization, Methodology, Software, Investigation, Formal Analysis, Writing - Original Draft. All authors contributed to the article and approved the submitted version.
